# Author’s reply to “A questionnaire-based cross-sectional study on neuropathic pain in patients with cancer in Japan: a deeper look into potential confounding variables”

**DOI:** 10.1093/jjco/hyaf151

**Published:** 2025-09-27

**Authors:** Saori Hashiguchi

**Affiliations:** Department of Palliative Medicine, St. Marianna University School of Medicine, 2-16-1 Sugao, Miyamae-ku, Kawasaki 216-8511, Japan

*To the Editor*:

We sincerely appreciate your interest in our manuscript [1] and taking the time to provide your highly insightful comments.

As you pointed out, we recognize that the survey method used in this study had limitations in definitively establishing the diagnosis of cancer-related neuropathic pain. Our study was based on a single questionnaire survey, completed by the patients themselves. Given the variability in patients’ medical knowledge, we judged that it would not be appropriate to analyze and discuss medical history and comorbidities such as hypertension or diabetes mellitus based on self-reports from patients. However, per Q7 of the survey, most patients (77.3%) reported that they did not have pain due to an injury or disease other than cancer. Therefore, while we cannot completely rule out the potential confounding influence of other comorbidities, we believe the collected data to be sufficiently applicable for the purposes of this study.

Additionally, questions regarding long-term treatment may not yield appropriate responses from patients given the design of this study. We hope that the points you raised, which we are not able to confirm in this survey, will serve as motivation for healthcare professionals who read this paper and are interested in initiating further research using more rigorous methods from a specialist’s perspective.

Regarding the lack of information on interventions such as physical therapy, cognitive-behavioral therapy, and alternative therapies, we were limited in how these could be investigated for similar reasons we outlined above; i.e. patients with varying levels of medical knowledge being asked to complete a single questionnaire survey. The focus of our study was to investigate the proportion of patients with potential cancer-related neuropathic pain under real-world clinical practice conditions, regardless of the type of adjunct therapies they may have been receiving. Regarding specific factors contributing to the onset of neuropathic pain in cases where comprehensive treatment is administered, we agree that further research will be necessary to clarify the impact of such adjunct therapeutic approaches.

Finally, we appreciate your suggestion regarding other lifestyle-related survey items that could have added clarity to the results. Unfortunately, lifestyle-related variables, such as alcohol use and physical activity, were not collected in the survey, so we were unable to control for these. We recognize that there are limitations to the survey items and interpretation of results using this survey method, but hope that our study sheds light on the issue of cancer-related pain from the patient perspective. We believe that future observational research could benefit from a more holistic approach in investigating lifestyle-related factors that could play a role in neuropathic pain, the results of which could potentially lead to better screening methods, earlier diagnosis, and ultimately better patient outcomes. We also believe that future studies will be more accurate if they include objective medical judgments by physicians.
